# An unusual case of metastasis to the left side of the heart: a case report

**DOI:** 10.1186/1752-1947-5-23

**Published:** 2011-01-20

**Authors:** Bharadwaj Cheruvu, Praveena Cheruvu, Michael Boyars

**Affiliations:** 1 Department of Internal Medicine, University of Texas, 301 University Boulevard, Galveston, TX 77555, USA

## Abstract

**Introduction:**

Cardiac metastases are found in six to 20% of autopsies of patients with malignant neoplasm. The most common neoplasms that metastasize to the heart are malignant melanoma, lymphoma, and leukemia, but the relative numbers are greater with breast and lung cancers, reflecting the most common incidence of these cancers.

**Case presentation:**

A 60-year-old Hispanic man presented to our hospital after being transferred from an outside hospital for workup and evaluation of an adrenal mass of the abdomen and pelvis, found on computed tomography. His chief complaint upon admission was altered mental status. Physical examination was unremarkable. He was alert and oriented and had a dry and non-erythematous oropharynx, and bilateral diffuse wheezing on lung examination. Computed tomography of the chest showed multiple hypodense lesions in the left ventricular myocardium, suggestive of metastases. There were also tiny sub-centimeter nodular densities in the right upper and lower lobes. Adrenal glands contained hypodense lesions, which showed characteristic adenocarcinomatous malignant cells.

**Conclusion:**

Cancers which have metastasized to the heart are found in six to 20% of patients with malignant neoplasms. The right side of the heart is more commonly involved in metastasis. This study is unusual in that a tumor of an unknown primary origin had metastasized to the left side of the heart.

## Introduction

Cardiac metastases are found in six to 20% of autopsies of patients with malignant neoplasms [1]. The most common neoplasms that metastasize to the heart are malignant melanoma, lymphoma and leukemia, but the relative numbers are greater with breast and lung cancers, reflecting the most common incidence of these cancers [2]. Cardiac metastases are usually found late and are rarely seen as the first site of metastases [1]. The orders of frequency of neoplasms to metastasize to the layers of the heart are epicardium (75.5%), myocardium (38.2%) and endocardium (15.5%).

Some factors have been postulated for the infrequency of cardiac metastasis: the strong action of the myocardium, metabolic peculiarities of striated muscle, rapid blood flow through the heart, and lymph flow moving away from the heart [3]. Neoplasms can metastasize to the heart by one of four different pathways: lymphatic, hematogenous, direct extension and transvenous extension via the superior or inferior vena cava [4]. Lymphatic spread often gives rise to pericardial metastases. Hematogenous spread has a propensity to migrate to the myocardium. Malignant melanoma, lymphoma, leukemia, soft tissue sarcoma are some of the neoplasms that would spread via the hematogenous route. Tumors that are in close proximity to the heart, such as bronchial, breast, and esophagus, most often spread by direct extension and give rise to pericardial disease.

The inferior vena cava is a common path of extension to the right atrium from sub-diaphragmatic organs such as the kidney, liver and adrenals. It has been reported that in one percent of patients with renal cell carcinoma, metastasis is via the renal vein into the right atrium [4].

Cardiac involvement is not uncommon in lung cancer; occurring in up to 25% of autopsy cases [5]. In a study by Tamura *et al*. 23 out of 78 cases had cardiac involvement found during autopsy [6]. Despite significant mortality and morbidity associated with cardiac metastasis, it is normally diagnosed during autopsy. Lung cancers normally spread to the heart via two mechanisms; direct extension, or intra-cavitary diffusion via the pulmonary veins.

The right side of the heart is more commonly involved by metastasis. A few cancer cases that have metastasis to the left side of heart were reported in 1954 [7]. Recently a left heart metastasis was detected for which a primary tumor had not been found by computed tomography (CT) scan. The purpose of this report is to present this unusual case.

### Case presentation

A 60-year-old homeless Hispanic man was transferred from a hospital to the University of Texas Medical Branch (UTMB) at Galveston for workup and evaluation of an adrenal mass found on CT of his abdomen and pelvis. He presented with altered mental status. Past medical history, according to his records, revealed hypertension and alcohol abuse. No information could be elicited from our patient, as he only responded by mumbling, with no family members by his side. On physical examination, he was afebrile with a blood pressure of 163/90, pulse of 101, and oxygen saturation of 95% on room air. He was alert and oriented, and appeared to be cachectic with poor nutritional status. A dry and non-erythematous orophaynx was found on physical examination. On cardiovascular

examination, his heart beat was tachycardiac with normal first and second heart sounds with no murmurs, rubs or gallops. Bilaterally diffuse wheezing was heard throughout all his lung fields. His abdomen was soft to palpation.

**Figure 1:**
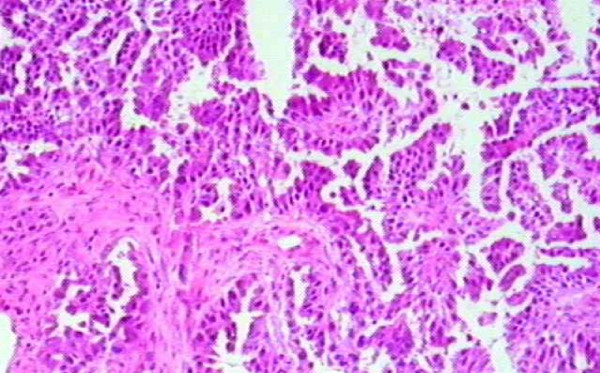
**CT guided aspiration of bilateral adrenals showing metastatic adenocarcinoma**.

**Figure 2:**
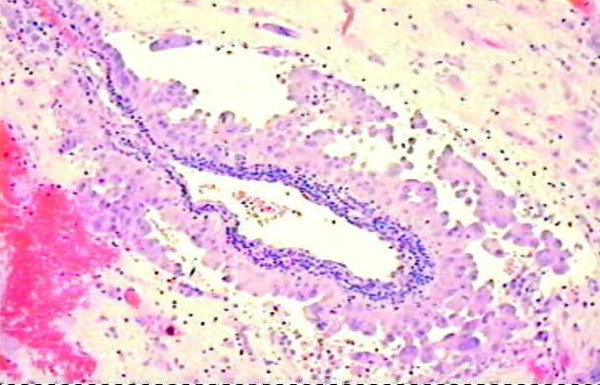
**CT guided biopsy of adrenal lesions, showing malignant adenocarcinoma cells**.

Our patient was slightly anemic with hemoglobin level of 11.9 with no leukocytosis. All electrolytes were within normal limits. A repeat CT scan was performed. CT of the chest showed mild calcified pleural thickening at the right apex. Tiny sub-centimeter nodular densities were detected in the right upper and lower lobes. The left ventricular myocardium contained multiple hypodense lesions which bore close resemblance to metastases. A centrally hypodense (likely necrotic), enlarged (size 2 cm) sub-carinal lymph node was also seen. The liver, spleen, pancreas, and kidneys appeared normal. Bilaterally, the adrenal glands contained large hypodense lesions measuring 2.6 × 2.3 cm on the right and 4.1 × 3 cm on the left. No intestinal wall thickening or evidence of intestinal mass was identified. Small, centrally hypodense nodular foci were seen in the abdomen and pelvis. Two were located in the peritoneum near the tip of the liver, and another was seen adjacent to the left acetabulum along the iliac chain. The latter bore close resemblance to a necrotic lymph node. Innumerable metastatic foci were seen throughout the posterior paraspinal musculature, iliopsoas muscles and gluteus muscles. A 1.2 cm metastatic lesion was seen in the left iliacus muscle superiorly and appeared to violate the anterior aspect of the left iliac wing. Sacrococcygeal metastatic lesion was also seen. A biopsy of the adrenal lesions performed under CT-guided aspiration showed characteristics of metastatic adenocarcinomatous malignant cells (Figures 1 and 2).

Trans-esophageal echo was performed to evaluate structural abnormality of the heart. There was moderate left ventricular hypertrophy. The left ventricular ejection fraction was estimated at 50 to 55%. Overall left ventricular systolic function was normal, but there was diastolic dysfunction consistent with impaired relaxation. The left atrium was enlarged. The aorta was normal. There was a moderate sized pericardial effusion with prominence in the anterior aspect of the right ventricle, but there was no echocardiographic evidence of tamponade.

Soon afterwards, our patient died. His family decided not to pursue an autopsy. Because of the short amount of time, workup for the primary cancer including colonoscopy and prostate-specific antigen screening was not performed.

## Discussion

Based on the physical and radiological findings, it is clear that this is a case of cancer with metastasis to multiple organs, such as the heart, bilateral adrenals, multiple musculoskeletal structures, lymph nodes, and peritoneum. CT scan of the chest, abdomen, and pelvis was performed to locate the primary site. In our patient, CT showed multiple hypodense lesions in the myocardium. Past history to rule in lung cancer, like tobacco abuse or occupational exposure was not available at this point. The radiologist recommended a six-month follow-up to further evaluate the lung lesions, but our patient died within the time frame. Biopsy of the adrenal mass was performed, which showed characteristics of metastatic adenocaricinoma.

Clinical presentation is normally related to that of the disseminated organ. However, most neoplasms that have metastasis to the heart are usually asymptomatic [8]. It was found that only 10% of patients with cardiac metastasis will have symptoms. The symptoms that are present would include tachycardia, arrhythmia, cardiomegaly or heart failure. Even though our patient was not able to admit to any symptoms, physical examination had revealed that he was tachycardic. The clinical manifestation varies depending upon the location and extent of myocardial involvement.

Diagnosis of cardiac metastasis is done primarily with use of echocardiography and imaging techniques (i.e. CT or magnetic resonance imaging (MRI)), as in our patient [9]. Echocardiography can provide information about cardiac tumors with regard to size, location, and response to treatment [10]. CT and MRI provide valuable information in regards to anatomical definition for pre-operative planning. For metastasis to the right side of the heart, transesophageal echo-guided biopsy is used for histological analysis. Since cardiac metastases are often incurable, palliative therapy should be offered. Systemic chemotherapy is usually the most beneficial in this situation. In cases where the metastasis results in tamponade, or obstruction of blood flow, palliative surgery is often used to relieve symptoms. Palliative radiotherapy to the heart is rarely helpful, or indicated to relieve symptoms.

A study by Young *et al*. in 1954 reported 13 cases of metastases to the heart out of 91 cancer cases [7]. The most common cancers were malignant lymphoma (61%), bronchial carcinoma (36.7%) and renal carcinoma (26.7%), as shown in Table 1. Another study by Berge *et al*. in 1968 reported 27 cases with metastases to the heart out of 122 cases of cancer [11]. The most common primary tumors were in the lung (16%), malignant lymphoma (14%), and kidney (11%). In the above studies, the primary tumors for left and right heart were not separated.

**Table 1:** Tumor types with heart metastasis (Young, 1954) [7].

Primary site	Total cases	Metastasis to the heart	Percentage
Bronchus	109	40	36.7

Malignant melanoma	17	11	64.7

Malignant lymphoma	60	9	15

Pancreas	33	7	21.2

Esophagus	33	6	18.2

Kidney	15	4	26.7

Testicle	9	4	44.4

Stomach	34	2	5.9

Prostrate	27	2	7.4

Bladder	15	1	

Larynx	11	1	

Skin	5	1	

Adrenal	3	1	

Paranasal sinus	2	1	

Lip	2	1	

A study by Alexandreseu *et al*. in 2009 reported a patient with metastatic adenocarcinoma of the right lower lobe to the right atrium [12]. Repeat CT after two rounds of chemotherapy had revealed a non-homogenous mass within the superior vena cava.

Recently two case reports have been published that presented with adenocarcinoma from the colon which have metastasized to the heart. In a study by Choi *et al*. a patient had presented to hospital with complaints of shortness of breath and weight loss [3]. A colonoscopy revealed adenocarcinoma of the sigmoid colon. Echocardiography revealed a mass in the right atrium, which revealed adenocarcinoma from the primary lesion. In a study by Bernhardt *et al*. a patient presented for artificial hip replacement. The patient also complained of occasional palpitations. Echocardiography revealed a mass in the left atrium [13]. MRI revealed a primary from gastric adenocarcinoma.

Klatt *et al*. in 1989 investigated 1095 malignancies in 1029 patients. They found eight adenocarcinomas of unknown primary. It was found that only two cases (25%) had metastasis to the heart [14]. This study makes it the only one of its kind which found two cases of unknown primary with metastasis to the heart.

Unlike many of the cases that were published in the literature, our case observed at UTMB had unknown primary adenocarcinoma which metastasized only to the left side of the heart. Our patient presented with no symptoms that suggested a source for the primary. He presented with symptoms related to the heart metastasis with tachycardia and wheezing. However extensive evaluation, i.e. colonoscopy, could not be carried out since our patient died.

The CT scan showed tiny sub-centimeter nodules in the lung that may be primaries, but, because of the size, it was difficult to ascertain whether these were primary or metastatic by biopsy. Also, it is very uncertain that metastasis to the heart, which usually occurs late in cancer, be caused by lung tumors so small.

## Conclusions

In conclusion, for patients with known metastatic neoplasms who present with cardiac symptoms, whether based on history or physical examination findings, the clinician should be alert for the possibility of cardiac metastases. Normally the symptoms are dependent on the location and extent of myocardial involvement. Cardiac involvement can be diagnosed with various imaging modalities, including trans-esophageal echocardiogram, CT and MRI. For patients with cardiac metastases, the options for treatment are limited to palliative treatment of symptoms and chemotherapy. In some cases, surgery is used to relieve symptoms.

## Consent

Written informed consent was obtained from the patient's next of kin for publication of this case report and any accompanying images. A copy of the written consent is available for review by the Editor-in-Chief of this journal.

## Competing interests

The authors declare that they have no competing interests.

## Authors' contributions

PC and MB analyzed and interpreted the patient's data. BC was a major contributor in writing the manuscript. All of the authors read and approved the final manuscript.
